# Identification of the Subcompartment-Specific Mitochondrial Proteome by APEX2 Proximity Labeling in *Saccharomyces cerevisiae*

**DOI:** 10.21769/BioProtoc.5605

**Published:** 2026-02-20

**Authors:** Lorenz Spänle, Johannes M. Herrmann

**Affiliations:** Cell Biology, University of Kaiserslautern, RPTU, Kaiserslautern, Germany

**Keywords:** *S. cerevisiae*, Mitochondria isolation, APEX2, Proximity labeling, Mitochondrial proteome, Sub-localization

## Abstract

The cellular compartments of eukaryotic cells are defined by their specific protein compositions. Different strategies are used for the identification of the subcellular proteomes, such as fractionation by differential centrifugation of cellular extracts. The localization of mitochondrial proteins is particularly challenging, as mitochondria consist of two membranes of different protein composition and two aqueous subcompartments, the intermembrane space (IMS) and the matrix. Previous studies identified subcompartment-specific proteomes by using combinations of hypotonic swelling and protease digestion followed by mass spectrometry. Here, we present an alternative, more unbiased method to identify the proteomes of mitochondrial subcompartments by use of an improved ascorbate peroxidase (APEX2) that is targeted to the IMS and the matrix. This method allows the subcompartment-specific labeling of proteins in mitochondria isolated from cells of the baker’s yeast *Saccharomyces cerevisiae*, followed by their purification on streptavidin beads. With this method, the proteins located in the different mitochondrial subcompartments of yeast cells can be efficiently and comprehensively identified.

Key features

• Coverage of ~75% of previous combined annotated mitochondrial proteome studies with high confidence in sub-localization probabilities.

• Provides detailed steps from starting culture to MS sample preparation, including the isolation of mitochondria.

• Allows for easy adaptations to compare different conditions and treatments.

• The whole experiment requires at least five days to complete.

## Graphical overview


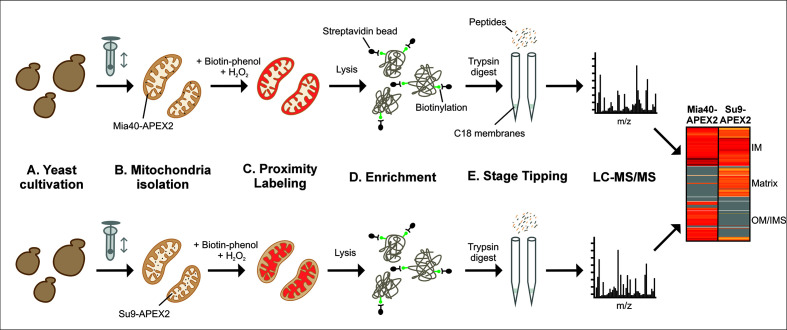


**Schematic overview of the subcompartment-specific labeling of mitochondrial proteins.** Yeast cells containing APEX2 in the intermembrane space (IMS) or matrix of their mitochondria are used. Mitochondria are isolated, and IMS (upper row) and matrix (lower row) proteins are biotinylated and identified by affinity purification and mass spectrometry (LC–MS/MS). The graph schematically illustrates the different stages of the procedure. IM, inner membrane; OM, outer membrane.

## Background

Mitochondria are essential organelles of eukaryotic cells. Four mitochondrial subcompartments can be distinguished: the outer membrane, the intermembrane space (IMS), the inner membrane, and the matrix. The mitochondrial proteome consists of about 900 (baker’s yeast) to 1,500 (human) different proteins [1,2], of which 40% are residents of the matrix, 40% of the inner membrane, and 10% each of the outer membrane and IMS.

A small number of mitochondrial proteins can be dually localized to different compartments [3]. The cytochrome b_5_ reductase of yeast mitochondria was the first protein described as dually localized to the outer and inner membranes [4]. Such dually localized proteins can function as signaling molecules that inform the health status of mitochondria. The best characterized example of such a sensor protein is PINK1, which, in poorly energized mitochondria, accumulates in the outer membrane to induce mitophagy [5]. Mutations in PINK1 were found in patients with familial forms of Parkinson’s disease [6]. The example of PINK1 clearly demonstrates that the intramitochondrial location of individual mitochondrial proteins can be of utmost relevance for (patho)physiology.

Several methods are used to determine the intramitochondrial localization of individual proteins. Microscopy, even high-resolution confocal microscopy, is often not well suited here, as the outer and inner membranes are only a few nanometers apart, so that proteins in the mitochondrial membranes and IMS are difficult to distinguish by imaging. Therefore, biochemical fractionation methods with isolated mitochondria, often in combination with protease treatment, are better suited; to open the outer, but not the inner membrane, hypotonic swelling or treatment with detergents such as digitonin can be used [7,8]. Alternatively, the outer membrane can be selectively opened by pro-apoptotic proteins [9]. Moreover, reporter strains had been developed, in which proteases (e.g., the tobacco etch virus protease) or split-GFP reporters target to the IMS or the matrix to detect the intramitochondrial location of individual tagged proteins [10].

An alternative, very powerful method for compartment-specific protein identification has been developed by the laboratories of Alice Ting and Vamsi Mootha, in which engineered versions of biotin ligases are targeted to the matrix and the IMS of mitochondria. This strategy proved to be extremely powerful for the identification of mitochondrial sub-proteomes in a global and unbiased manner [11,12]. For protein biotinylation, an engineered ascorbate peroxidase (APEX) was employed, which, upon incubation with biotin-phenol and hydrogen peroxide, forms highly reactive, short-lived radicals. These radicals lead to the covalent addition of biotin to the side chains of electron-rich amino acid residues such as tyrosine, tryptophan, histidine, or cysteine [13]. Since the generated radicals diffuse quickly within a compartment but cannot efficiently cross lipid bilayers, APEX-driven biotinylation (*APEX labeling*) is well-suited for compartment-specific proteome mapping, complementing other proximity labeling methods such as BioID or TurboID, which are typically used for the detection of interaction partners of proteins [14].

**Figure 1.**
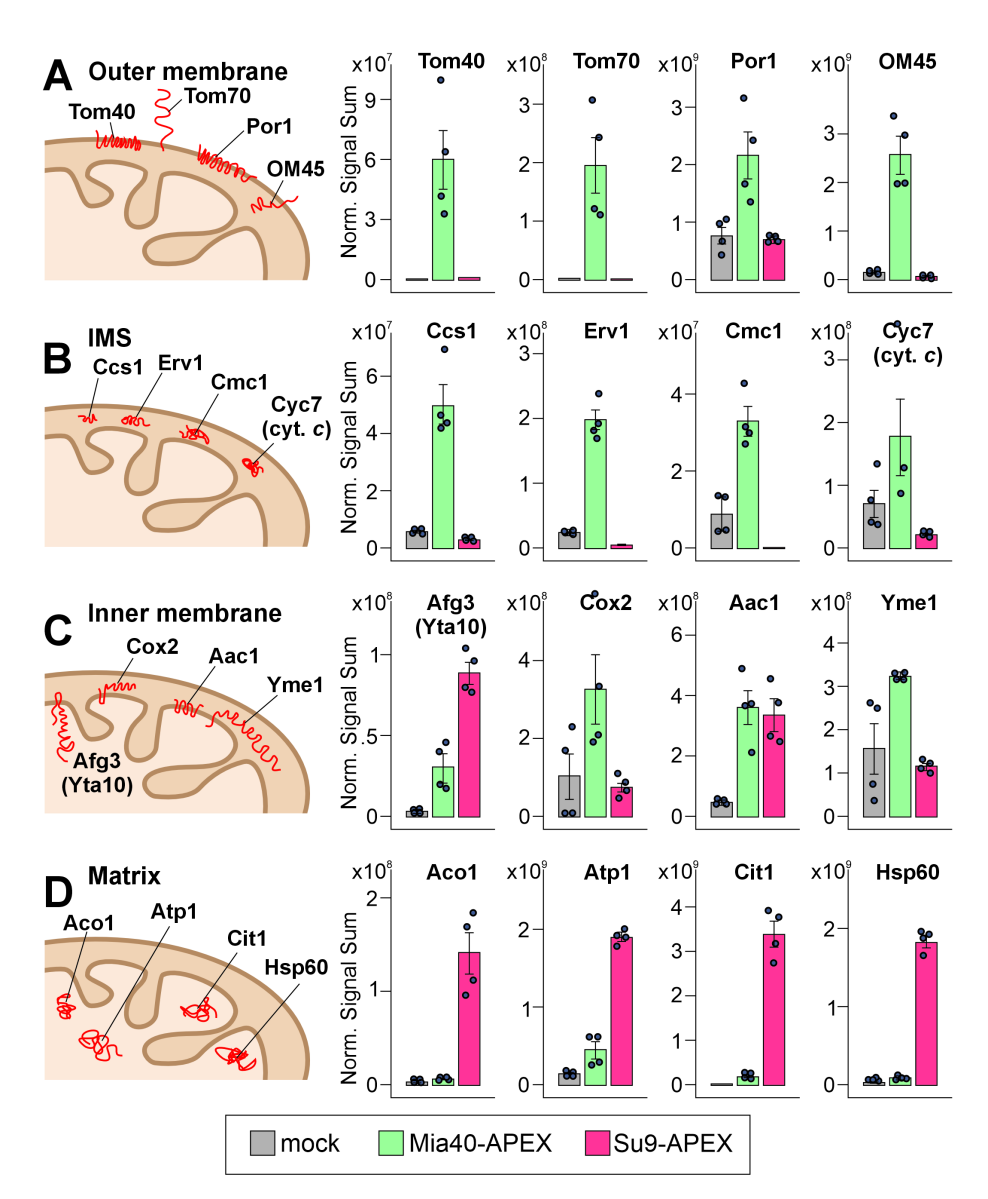
Ascorbate peroxidase (APEX2)-mediated biotinylating patterns reveal intramitochondrial distribution of proteins [15]. Protein levels were detected by mass spectrometry in the streptavidin pulldowns from lysed isolated mitochondria, derived from cells expressing no APEX (gray), Mia40-APEX (green), or Su9-APEX (red). Shown are the normalized signal sums of mitochondrial proteins of the four different mitochondrial subcompartments: (A) outer membrane, (B) intermembrane space (IMS), (C) inner membrane, and (D) matrix. Plotted are mean values and standard deviations from four replicates. The intramitochondrial localizations of the proteins are sketched on the left.

APEX labeling works very efficiently with mammalian cells owing to the high permeability of human membranes for biotin-phenol [12]. However, the cell wall of fungal and plant cells is not permeable to biotin-phenol, limiting the use of biotin-phenol in these systems. In this method paper, we describe how to use APEX2 labeling for the detection of subproteomes of yeast mitochondria, employing purified mitochondria as starting material [15]; to this end, we used APEX2, which is an improved APEX variant generated by directed evolution [13,16]. This method works extremely well and allows the quantitative detection of proteins in the IMS and matrix (Figure 1). Moreover, it can be used to determine the topology of inner proteins.

## Materials and reagents

**Biological materials**

1. *Saccharomyces cerevisiae* W303 strain (*MATα leu2-3,112 trp1-1 can1-100 ura3-1 ade2-1 his3-11,15* [phi+]) (Ralser et al. [17])

2. *Saccharomyces cerevisiae* W303 strain + Su9-APEX2-FLAG (*YPRC∆15-pRNR1- Mia40(1-70)-APEX2-FLAG-tTDH1-HygromycinR*) (see Appendix in [15])

3. *Saccharomyces cerevisiae* W303 strain + IMS-APEX2-FLAG (*YPRCD15-pRNR1-Su9-APEX2-FLAG-tTDH1-HygromycinR*) (see Appendix in [15])

*Note: Yeast strains and plasmids used for integration are available upon request.*

**Reagents**

1. Adenine hemisulfate salt (Sigma-Aldrich, catalog number: A3159)

2. L-Arginine (Sigma-Aldrich, catalog number: A8094)

3. L-Histidine monohydrochloride monohydrate (Sigma-Aldrich, catalog number: H5659)

4. L-Isoleucine (Sigma-Aldrich, catalog number: I7403)

5. L-Leucin (Sigma-Aldrich, catalog number: L8000)

6. L-Lysin HCl (Sigma-Aldrich, catalog number: L8662)

7. L-Methionine (Sigma-Aldrich, catalog number: M5308)

8. L-Phenylalanine (Sigma-Aldrich, catalog number: P2126)

9. L-Threonine (Sigma-Aldrich, catalog number: T8441)

10. L-Tryptophan (Sigma-Aldrich, catalog number: T8941)

11. L-Tyrosine (Sigma-Aldrich, catalog number: T8566)

12. Uracil (Sigma-Aldrich, catalog number: U1128)

13. L-Valine (Sigma-Aldrich, catalog number: V0513)

14. L-Proline (Sigma-Aldrich, catalog number: P5607)

15. Yeast-nitrogen-base (Formedium, catalog number: CYN0510)

16. Yeast-nitrogen-base w/o biotin (Formedium, catalog number: CYN3202)

17. Ammonium sulfate (Sigma-Aldrich, catalog number: 1.01217)

18. Galactose (Sigma-Aldrich, catalog number: G0625)

19. Dithiothreitol (DTT) (AppliChem, catalog number: A1101)

20. Sorbitol (Carl Roth, catalog number: 6213.2)

21. KH_2_PO_4_ (Bernd Kraft, catalog number: 15258.3600)

22. K_2_HPO_4_ (Carl Roth, catalog number: 7758-11-4)

23. Tris(hydroxymethyl)aminomethane (Serva, catalog number: 77-81-1)

24. HCl 37% (VWR Chemicals, catalog number: 20252.335)

25. Zymolyase (Carl Roth, catalog number: 9324.3)

26. EDTA disodium salt 2-hydrate (AppliChem, catalog number: 131669)

27. Albumin bovine fraction V, fatty acid free (BSA) (Serva, catalog number: 11932.03)

28. Phenylmethylsulphonyl fluoride (PMSF) (Carl Roth, catalog number: 6367.2)

29. Protein assay dye (Bio-Rad Laboratories, catalog number: 5000006)

30. Liquid N_2_

31. Biotin-phenol (Sigma-Aldrich, catalog number: SML2135)

32. Dimethyl sulfoxide (DMSO) (VWR Chemicals, catalog number: 23500.397)

33. H_2_O_2_ 30% (Sigma-Aldrich, catalog number: 216763)

34. Ascorbic acid (AppliChem, catalog number: A3604)

35. Sodium azide (Merck, catalog number: 8.22335)

36. Trolox (Thermo Scientific, catalog number: 218940050)

37. NaH_2_PO_4_·H_2_O (Merck, catalog number: 1.06346)

38. Na_2_HPO_4_ (Sigma-Aldrich, catalog number: 71640)

39. NaCl (Fisher Scientific, catalog number: 10616082)

40. IGEPAL CA-630 (Sigma-Aldrich, catalog number: I8896)

41. Deoxycholic acid sodium salt (Carl Roth, catalog number: 3484.2)

42. Sodium dodecyl sulfate (SDS) (Serva, catalog number: 20765.03)

43. Protease inhibitor (cOmplete tablets) (Roche, catalog number: 04693159001

44. Pierce streptavidin magnetic beads (Thermo Scientific, catalog number: 88817)

45. Urea (Serva, catalog number: 24524.0)

46. Trypsin (Promega, catalog number: V5111)

47. Trypsin resuspension buffer (Promega, catalog number: V5111)

48. Chloroacetamide (CAA) (Sigma-Aldrich, catalog number: C0267)

49. Formic acid (Honeywell, catalog number: 94318)

50. Acetonitrile (VWR Chemicals, catalog number: 83640.290)

51. MS-grade H_2_O (VWR Chemicals, catalog number: 23595.328)

52. Trifluoroacetic acid (TFA) (Carl Roth, catalog number: 6957.1)

53. Methanol (Honeywell, catalog number: 154903)

54. HEPES (AppliChem, catalog number: A1069)

55. NaOH (Sigma-Aldrich, catalog number: 06203)

**Solutions**

1. Drop-out-mix, 20× (DoM) (see Recipes)

2. Galactose, 30% (see Recipes)

3. S, 5× (see Recipes)

4. S-biotin, 5× (see Recipes)

5. SGal medium (see Recipes)

6. SGal medium lacking biotin (SGal-biotin medium) (see Recipes)

7. MP1 buffer (see Recipes)

8. Sorbitol, 2.4 M (see Recipes)

9. Sorbitol, 1.2 M (see Recipes)

10. KH_2_PO_4_, 1 M (see Recipes)

11. K_2_HPO_4_, 1 M (see Recipes)

12. KPi buffer, 1 M, pH 7.4 (see Recipes)

13. MP2 buffer (see Recipes)

14. Tris, 1 M, pH 7.4 (see Recipes)

15. Tris, 20 mM (see Recipes)

16. EDTA, 0.5 M, pH 8.0 (see Recipes)

17. Homogenization buffer (HB) (see Recipes)

18. HEPES, 1 M, pH 7.4 (see Recipes)

19. SH buffer (see Recipes)

20. Biotin-phenol, 5.5 mM (see Recipes)

21. H_2_O_2_, 100 mM (see Recipes)

22. Ascorbic acid, 250 mM (see Recipes)

23. Sodium azide, 1% (see Recipes)

24. Trolox, 100 mM (see Recipes)

25. Quencher cocktail (see Recipes)

26. NaPi buffer, 200 mM, pH 7.4 (see Recipes)

27. NaCl, 5 M (see Recipes)

28. Sodium dodecyl sulfate (SDS), 10% (see Recipes)

29. Protease inhibitor, 10× (see Recipes)

30. Lysis buffer (RIPA) (see Recipes)

31. Urea, 8 M (see Recipes)

32. Trypsin, 0.5 μg/μL (see Recipes)

33. Dithiothreitol (DTT), 1 M (see Recipes)

34. Elution buffer I (see Recipes)

35. Chloroacetamide (CAA), 400 mM (see Recipes)

36. Elution buffer II (see Recipes)

37. Trifluoroacetic acid (TFA), 10% (see Recipes)

38. Buffer A (see Recipes)

39. Buffer B (see Recipes)

40. Buffer C (see Recipes)

**Recipes**

**1. Drop-out-mix, 20× (DoM)**

**Table:** 

Reagent	Final concentration	Quantity or volume
Adenine hemisulfate salt	2.2 mM	400 mg
L-Arginine	2.3 mM	400 mg
L-Histidine HCL monohydrate	1.9 mM	400 mg
L-Isoleucine	4.6 mM	600 mg
L-Leucin	15.3 mM	2,000 mg
L-Lysin HCl	3.3 mM	600 mg
L-Methionine	2.7 mM	400 mg
L-Phenylalanine	6.1 mM	1,000 mg
L-Threonine	33.3 mM	4,000 mg
L-Tryptophan	2.0 mM	400 mg
L-Tyrosine	2.2 mM	400 mg
Uracil	3.6 mM	400 mg
L-Valine	25.6 mM	3,000 mg
L-Proline	34.8 mM	4,000 mg
Double-distilled water (ddH_2_O)	n/a	1 L
Total	n/a	1 L

Autoclave at 120 °C for 20 min.

**2. Galactose, 30%**

Dissolve 30 g of galactose in up to 100 mL of ddH_2_O by stirring and heating up. Autoclave at 120 °C for 20 min.

**3. S, 5×**

**Table:** 

Reagent	Final concentration	Quantity or volume
Yeast-nitrogen-base	5×	9.5 g
Ammonium sulfate	189.2 mM	25 g
ddH_2_O	n/a	1 L
Total	n/a	1 L

Autoclave at 120 °C for 20 min.

**4. S-biotin, 5×**

**Table:** 

Reagent	Final concentration	Quantity or volume
Yeast-nitrogen-base w/o biotin	1×	9.5 g
Ammonium sulfate	189.2 mM	25 g
ddH_2_O	n/a	1 L
Total	n/a	1 L

Autoclave at 120 °C for 20 min.

**5. SGal medium**

**Table:** 

Reagent	Final concentration	Quantity or volume
5× S	1×	200 mL
20× DoM	1×	50 mL
30% galactose	2%	67 mL
ddH_2_O (autoclaved)	n/a	683 mL
Total	n/a	1 L

Mix reagents in an autoclaved bottle.

**6. SGal-biotin medium**

**Table:** 

Reagent	Final concentration	Quantity or volume
5× S-biotin	1×	200 mL
20× DoM	1×	50 mL
30% galactose	2%	67 mL
ddH_2_O (autoclaved)	n/a	883 mL
Total	n/a	1 L

Autoclave at 120 °C for 20 min.

**7. MP1 buffer**

**Table:** 

Reagent	Final concentration	Quantity or volume
Tris	100 mM	12.11 g
Dithiothreitol	10 mM	154.25 mg
ddH_2_O	n/a	100 mL
Total	n/a	1 L

**8. Sorbitol, 2.4 M**

Dissolve 43.72 g of sorbitol in ddH_2_O to a final volume of 100 mL. Autoclave at 120 °C for 20 min.

**9. Sorbitol, 1.2 M**

Dissolve 21.86 g of sorbitol in ddH_2_O to a final volume of 100 mL. Autoclave at 120 °C for 20 min.

**10. KH_2_PO_4_, 1 M**

Dissolve 13.61 g of KH_2_PO_4 _in ddH_2_O to a final volume of 100 mL.

**11. K_2_HPO_4_, 1 M**

Dissolve 171.8 g of K_2_HPO_4_ in ddH_2_O to a final volume of 1 L.

**12. KPi buffer, 1 M, pH 7.4**

Mix 50 mL of 1 M KH_2_PO_4_ with ~525 mL of 1 M K_2_HPO_4_ to adjust to pH 7.4.

**13. MP2 buffer**

**Table:** 

Reagent	Final concentration	Quantity or volume
2.4 M sorbitol	1.2 M	50 mL
1 M KPi buffer pH 7.4	20 mM	2 mL
ddH_2_O	n/a	48 mL
Total	n/a	100 mL

**14. Tris, 1 M, pH 7,4**

Dissolve 121.1 g of Tris(hydroxymethyl)aminomethane in ~800 mL of ddH_2_O and adjust the pH to 7.4 by adding 37% HCl dropwise. Fill up to 1 L with ddH_2_O.

**15. Tris, 20 mM, pH 7.4**

Mix 20 mL of 1 M Tris pH 7.4 with 980 mL of ddH_2_O.

**16. EDTA, 0.5 M, pH 8.0**

Dissolve 18.6 g of EDTA disodium salt 2-hydrate in ddH_2_O to a final volume of 100 mL. Adjust pH to 8.0 with NaOH.

**17. Homogenization buffer (HB)**

**Table:** 

Reagent	Final concentration	Quantity or volume
1 M Tris pH 7.4	10 mM	2 mL
0.5 M EDTA pH 8.0	1 mM	0.4 mL
BSA	0.2%	400 mg
PMSF	1 mM	34.84 mg
2.4 M sorbitol	0.6 M	50 mL
ddH_2_O	n/a	147.6 mL
Total	n/a	200 mL

**Critical:** Add PMSF and BSA fresh and just before use.

**18. HEPES, 1 M, pH 7.4**

Dissolve 23.8 g of HEPES in ddH_2_O to a final volume of 100 mL. Adjust to pH 7.4 with NaOH.

**19. SH buffer**

**Table:** 

Reagent	Final concentration	Quantity or volume
2.4 M sorbitol	0.6 M	25 mL
1 M HEPES pH 7.4	20 mM	2 mL
ddH_2_O	n/a	73 mL
Total	n/a	100 mL

**20. Biotin-phenol, 5.5 mM**

Dissolve 2 mg of biotin-phenol in DMSO to a final volume of 1 mL.

**21. H_2_O_2_, 100 mM**

Dilute 5 μL of 30% H_2_O_2 _in 495 μL of ddH_2_O.

**22. Ascorbic acid, 250 mM**

Dissolve 44 mg of ascorbic acid in ddH_2_O to a final volume of 1 mL.

**23. Sodium azide, 1%**

Dissolve 100 mg of sodium azide in 9.9 mL of ddH_2_O.

**24. Trolox, 100 mM**

Dissolve 25 mg of Trolox in ddH_2_O to a final volume of 1 mL.

**25. Quencher cocktail**

**Table:** 

Reagent	Final concentration	Quantity or volume
250 mM ascorbic acid	10 mM	400 μL
1% sodium azide	10 mM	660 μL
100 mM Trolox	5 mM	500 μL
SH buffer	n/a	8.44 mL
Total	n/a	10 mL

**26. NaPi buffer, 200 mM, pH 7.4**

Dissolve 0.47 g of NaH_2_PO_4_·H_2_O and 2.355 g of Na_2_HPO_4_ in ddH_2_O to a final volume of 100 mL.

**27. NaCl, 5 M**

Dissolve 58.44 g of NaCl in ddH_2_O to a final volume of 200 mL.

**28. SDS, 10%**

Dissolve 10 g of SDS in up to 100 mL of ddH_2_O.

**29. Protease inhibitor, 10×**

Dissolve 1 tablet of protease inhibitor in 1 mL of ddH_2_O.

**30. RIPA**

**Table:** 

Reagent	Final concentration	Quantity or volume
200 mM NaPi buffer pH 7.4	20 mM	10 mL
5 M NaCl	150 mM	3 mL
IGEPAL CA-630	1%	1 mL
Deoxycholic acid sodium salt	0.5% (w/v)	500 mg
10% SDS	0.1%	1 mL
10× protease inhibitor	1×	10 mL
ddH_2_O	n/a	75 mL
Total	n/a	100 mL

**31. Urea, 8 M**

Dilute 96.1 g of urea in MS-grade ddH_2_O to a final volume of 200 mL.

**32. Trypsin, 0.5 μg/μL**

Dissolve 20 μg of trypsin (1 ampule) in 40 μL of trypsin resuspension buffer. To activate the trypsin, incubate at 30 °C for 15 min. Leftover trypsin solution can be stored at -80 °C.

**33. DTT, 1 M**

Dissolve 154 mg of DTT in MS-grade ddH_2_O to a final volume of 1 mL

**34. Elution buffer I**

**Table:** 

Reagent	Final concentration	Quantity or volume
8 M urea	2 M	250 μL
1 M Tris pH 7.4	50 mM	50 μL
1 M DTT	1 mM	1 μL
0.5 μg/μL trypsin	5 ng/μL	10 μL
MS-grade ddH_2_O	n/a	689 μL
Total	n/a	1 mL

**35. CAA, 400 mM**

Dissolve 37.4 mg of CAA in MS-grade ddH_2_O to a final volume of 1 mL.

**36. Elution buffer II**

**Table:** 

Reagent	Final concentration	Quantity or volume
8 M urea	2 M	250 μL
1 M Tris pH 7.4	50 mM	50 μL
400 mM CAA	5 mM	25 μL
0.5 μg/μL trypsin	5 ng/μL	10 μL
MS-grade ddH_2_O	n/a	665 μL
Total	n/a	1 mL

**37. TFA, 10%**

Dissolve 5 mL of TFA in 45 mL of MS-grade ddH_2_O.

**38. Buffer A**

Dissolve 50 μL of formic acid in 50 mL of MS-grade ddH_2_O.

**39. Buffer B**

Mix 50 μL of formic acid, 40 mL of acetonitrile, and 10 mL of MS-grade ddH_2_O.

**40. Buffer C**

Mix 100 μL of 10% TFA, 10 μL of formic acid, and 10 mL of MS-grade ddH_2_O.

**Laboratory supplies**

1. 1.5 mL microtubes (Eppendorf, catalog number: 0030121023)

2. pH-indicator paper (Acilit, Merck, catalog number: 1.09560.0003)

3. Solid-phase extraction disk (C18 membrane) (Supelco, catalog number: 66883-U)

4. epT.I.P.S. (MS stage tips) (Eppendorf, catalog number: 022491539)

5. Erlenmeyer flasks (100, 500, 3,000 mL)

6. Pipette tips (10, 200, 1,000 μL)

7. Glass pipettes (5, 10, 20 mL)

8. Falcons (15, 50 mL)

9. Plastik pipette (5 mL)

10. Cuvettes

11. Ice

## Equipment

1. Cell density meter (Fisher Scientific, model: Harvard Biochrom Ultrospec^TM^ 10, catalog number: 10704417)

2. High-performance centrifuge (Beckman Coulter, model: Avanti J-26 XP, catalog number: B42205)

3. 500 mL centrifugation bottles (Beckman Coulter, catalog number: 355607)

4. Dounce homogenizer plunger + cylinder (Sartorius, catalog numbers: 8530750, 8530939)

5. UV/Visible spectrophotometer (Biochrom, model: Ultrospec 2100 pro)

6. Pipette controller (Sigma-Aldrich, model: Hirschmann pipetus, catalog number: Z314951)

7. Cell disruptor (Scientific Industries, model: Disruptor genie, catalog number: D238)

8. Magnetic rack (Thermo Fisher, catalog number: 12321D)

9. Thermo shaker (Eppendorf, model: ThermoMixer C, catalog number: 5382000015)

10. Tube rotator (Stuart Scientific, model: SB1)

11. Spring-loaded syringe [18]

12. Stage tipping centrifuge (Fisher Scientific, model: Sonation, catalog number: NC2824232)

13. In-house 3D-printed frame to elute the stage tips (Figure 2) [19]

**Figure 2.**
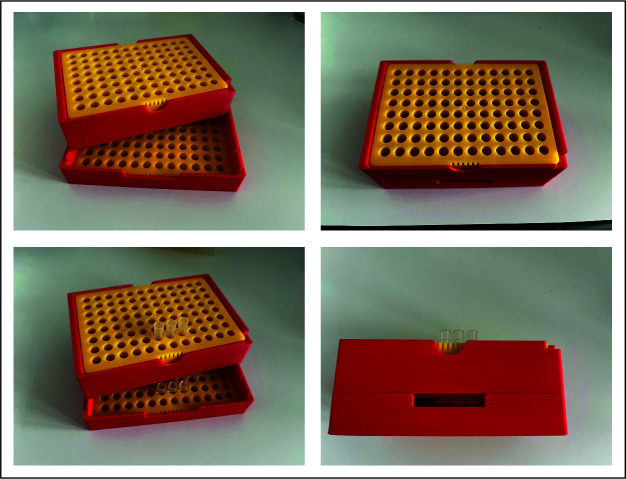
Model of the in-house 3D-printed frame. This model places the loaded stage tips over 200 μL reaction tubes. It enables elusion by centrifugation in a Beckman JS-5.3 rotor [19].

14. Vacuum centrifuge (Eppendorf, model: Concentrator plus, catalog number: 5305000509)

15. Scale

16. Polystyrene box

17. Dewar flask

18. Incubator

## Procedure

**A. Yeast cultivation**

1. Inoculate a fresh colony of *S. cerevisiae* in 20 mL of liquid SGal medium and incubate overnight at 130 rpm and 30 °C.

*Note: The experiment is performed with biological replicates. Use four different starting colonies for each strain and condition.*

2. Dilute the overnight culture in 200 mL of liquid SGal medium to an optical density of 0.2 at 600 nm (OD_600_) using the Ultrospec^TM^ cell density meter. Incubate the culture for 8 h at 130 rpm and 30°C.

3. Dilute the culture in 2 L of liquid SGal-biotin medium to an OD_600_ of 0.025 and incubate overnight (18 h) at 130 rpm and 30 °C.

*Note: These dilutions are based on the growth behavior of the cells. Adapt the dilutions to maintain exponential growth before starting the mitochondria isolation in the morning, as they may vary.*

**B. Mitochondria isolation**

*Note: A visual representation of the procedure can be found in [20].*

1. Distribute the culture in six 500 mL centrifugation bottles to harvest cells in the exponential phase with a Beckman Coulter Avanti J-26 XP (rotor: JA10) at 2,800× *g* for 5 min.

2. Measure the weight of an empty bottle (W1).

3. Discard the supernatant and wash the cell pellets with ~15 mL of ddH_2_O. Transfer the resuspended pellets to the previously weighed bottle and spin down at 2,800× *g* for 5 min.

4. Remove the supernatant and determine the wet weight (WW) of the cell pellet by weighing the bottle again (W2 - W1 = WW).

5. Resuspend the pellet in MP1 buffer (2 mL per g WW) and incubate for 10 min at 130 rpm and 30 °C.

6. Centrifuge at 2,800× *g* for 5 min and remove supernatant.

7. Wash the pellet in 50 mL of 1.2 M sorbitol, centrifuge at 2,800× *g* for 5 min, and remove the supernatant.

8. Dissolve 3 mg/g WW zymolyase in the MP2 buffer (6.7 mL/g WW) and resuspend the cell pellet with the prepared MP2 buffer. Incubate the cell solution for 60 min at 130 rpm and 30 °C.

**Critical:** Cool down the centrifuge to 4 °C and work only on ice from now on.

9. Centrifuge at 2,200× *g* for 5 min and remove supernatant.

10. Dissolve and resuspend the pellet in half of the volume of HB (13.4 mL/g WW) and transfer to a cooled Dounce homogenizer.

11. Place the Dounce homogenizer on ice and homogenize the cell solution by gently moving the plunger up and down 15 times.

12. Pour the homogenate back into the bottle and wash the homogenizer with the leftover HB.

**Critical:** The next step is to collect the supernatant and not discard it!

**Figure 3.**
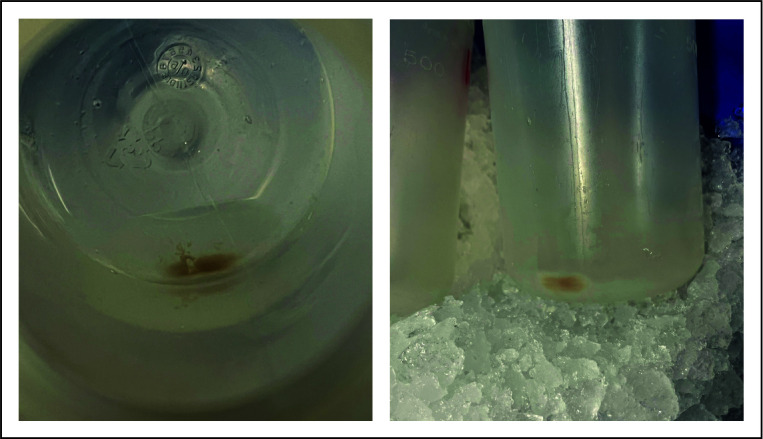
Image of the mitochondrial pellet during mitochondrial isolation. Visible is a small mitochondrial pellet at the bottom of a 500 mL Beckmann centrifugation bottle. The pellet was obtained after centrifugation at 17,500× *g* for 12 min. The picture was taken before washing with SH buffer. The size of the mitochondrial pellet is the result of a 2 L starting culture.

13. Centrifuge at 2,800× *g* for 5 min at 4 °C to spin down cell debris. Transfer the supernatant to a fresh bottle. Discard the pellet.

14. Repeat the previous step for as long as there is a visible pellet.

15. Centrifuge the supernatant at 17,500× *g* for 12 min and resuspend the pellet (mitochondria) in 5 mL of cold SH buffer. Use cut tips or a plastic pipette with bigger openings (see Figure 3).

16. Centrifuge at 17,500× *g* for 12 min and remove supernatant.

17. Resuspend the pellet in 300 μL of SH buffer.

18. Take an aliquot to determine protein concentration with a protein assay dye and a UV/Vis spectrometer (Bradford assay).

19. Dilute the mitochondrial suspension to a final concentration of 10 mg/mL with SH buffer.

20. Snap-freeze 50 μL aliquots in liquid nitrogen and store at -80 °C.

**Pause point:** Mitochondrial aliquots can be stored at -80 °C for more than one year.

**C. Proximity labeling**

1. Thaw 50 μL of mitochondrial suspension (10 mg/mL) on ice.

2. Add 38.2 μL of SH buffer and 1.8 μL of 5.5 mM biotin-phenol and incubate mitochondria for 30 min at 900 rpm and 30 °C.

3. Add 1 μL of 30% H_2_O_2_, vortex at low speed, and incubate for 5 min at 30 °C.

4. Add 100 μL of ice-cold quencher cocktail and centrifuge at 25,000× *g* for 10 min at 4 °C. Remove the supernatant.

5. Wash with 100 μL of quencher cocktail and centrifuge at 25,000× *g* for 10 min at 4 °C. Remove the supernatant.

6. Resuspend the pellet in 500 μL of ice-cold lysis buffer (RIPA).

7. Use the cell disruptor for 15 min at 4 °C to break up the mitochondrial membrane.

8. Centrifuge the lysate at 17,000× *g* for 10 min at 4 °C. Transfer the supernatant into a fresh tube.

**D. Enrichment**

1. Wash 40 μL of streptavidin beads 2× with 500 μL of RIPA buffer. Remove the supernatant with a magnetic rack.

2. Resuspend washed beads in 40 μL of RIPA buffer and add the mitochondrial lysate.

3. Incubate for 1 h tumbling at 4 °C.

4. Separate the beads on a magnetic rack and discard the supernatant.

5. Wash the beads 2× with 250 μL of RIPA buffer and 1× with 250 μL of 20 mM Tris buffer.

6. Again, add 250 μL of 20 mM Tris buffer and transfer everything to a fresh tube to remove excess detergent.

7. Add 50 μL of elution buffer I and incubate for 1 h at room temperature (RT) with tumbling.

8. Separate beads on a magnetic rack and transfer supernatant to a fresh tube.

9. Add 50 μL of elution buffer II to the beads and incubate for 30 min at RT with tumbling.

10. Separate beads on a magnetic rack, transfer supernatant, and combine with the first elution.

11. Continue the digest of the elution overnight (16 h) in the dark at 37 °C.

**E. Stage tipping and MS**

*Note: See General note 1.*

1. Acidify digested peptides by adding 10 μL of 10% TFA and spin down the sample at 13,000× *g* for 1 min.

2. Use 3× C18 membrane layers to purify peptides. Punch the C18 membranes with a spring-loaded syringe and transfer them to a pipette tip [18].

3. Equilibrate the tips with 100 μL of methanol and wash 2× with 100 μL of buffer A. Always spin down the volume using a stage tipping centrifuge.

4. Load acidified peptides (100 μL) onto the stage tip.

5. Wash the peptides on the C18 membrane with 100 μL of buffer A.

**Pause point:** The peptide-loaded tips can be stored in the fridge for some weeks.

6. Elute peptides with 40 μL of buffer B by transferring them to a 200 μL Eppendorf tube at 500× *g* for 5 min.

*Note: We used an in-house 3D-printed frame to place the stage tips over the Eppendorf tubes (Figure 2).*

7. Evaporate the sample in a vacuum centrifuge for 2 h at RT.

8. Add 9 μL of buffer C. The sample is ready for LC–MS/MS.

## Validation of protocol

This protocol has been used and validated in the following research article (Figure 1):

• Flohr et al. [15]. Dysfunctional mitochondria trap proteins in the intermembrane space. *EMBO J*. (2025), 44: 4352–4377, DOI: https://doi.org/10.1038/s44318-025-00486-1

## General notes and troubleshooting

**General notes**

1. Solid-phase extraction (SPE) was performed using C18 stage tips prepared in-house by a spring-loaded syringe and an available 3D-printed frame. Alternatively, commercially available C18 spin columns or 96-well disks can be used (e.g., by preomics). The design files for the 3D-printed frame are available upon request from the authors.

2. To monitor the degree of cell wall digestion with the MP2 buffer, combine 50 μL of cell solution with 2 mL of water and 2 mL of 1.2 M sorbitol. The OD_600_ should be lower in water due to the eruption of cells without cell walls.

3. This method was verified in a W303 yeast strain. We assume that this method is also applicable to other commonly used yeast lab-strains (e.g., BY4741).

**Troubleshooting**

1. There is no or just a small mitochondrial pellet: Using 2 L of cell suspension at 1 OD_600_ is normally sufficient as starting material for a decent number of mitochondria. Different conditions or mutations can change the mitochondrial volume. To fix the problem, increase the number of cells.
